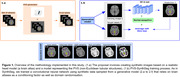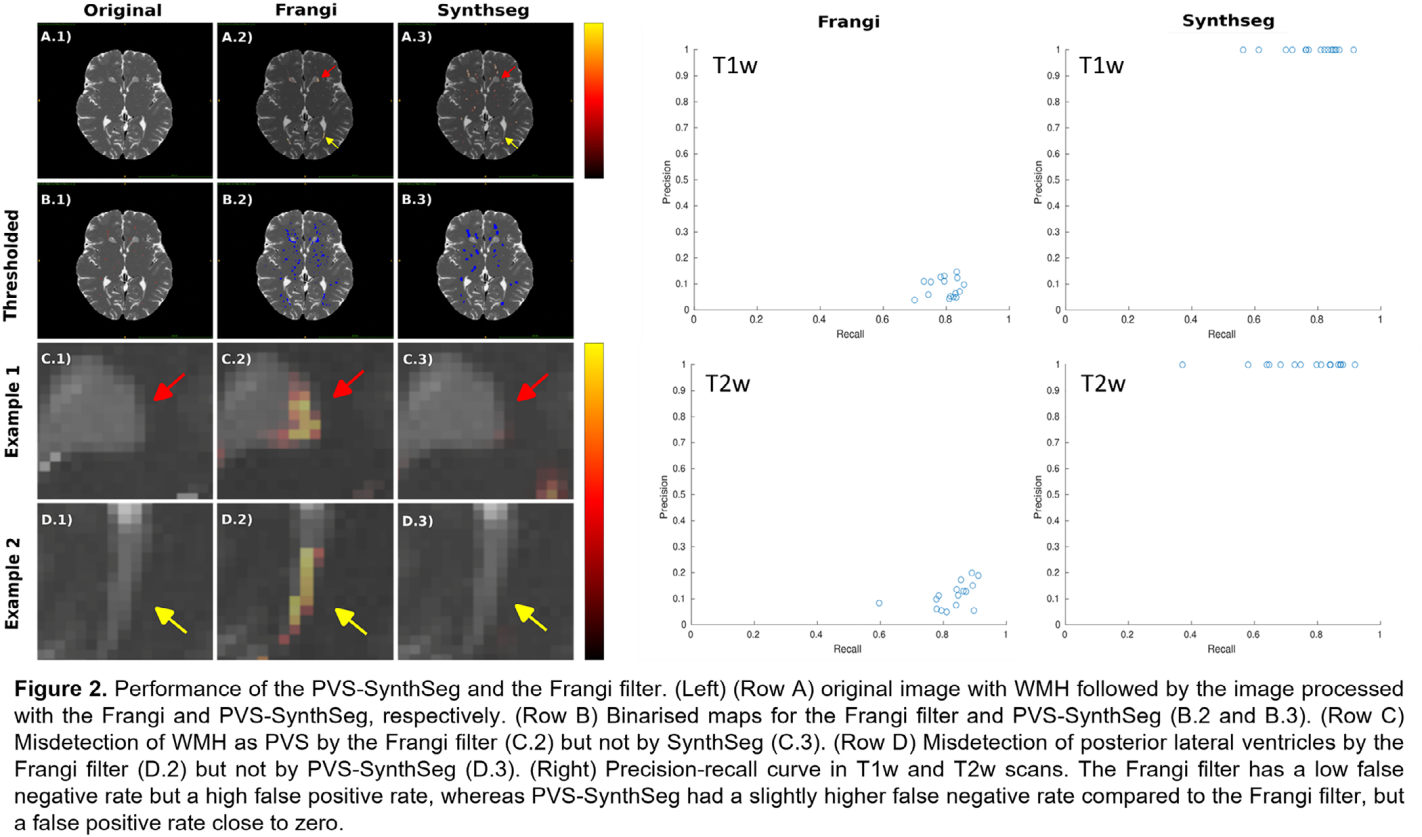# Contrast‐agnostic deep‐learning‐based detection of perivascular spaces in magnetic resonance imaging

**DOI:** 10.1002/alz.088012

**Published:** 2025-01-09

**Authors:** Mario Díaz, Erelle Fuchs, Hendrik Mattern, Daniel Behme, Roberto Duarte, Maria del C. Valdes Hernandez, Joanna M Wardlaw, Stefanie Schreiber, Maria Trujillo, Jose Bernal Moyano

**Affiliations:** ^1^ Multimedia and Computer Vision Group, Universidad del Valle, Cali, Valle del Cauca Colombia; ^2^ Department of Neuroradiology, University Hospital Magdeburg, Magdeburg, Sachsen‐Anhalt Germany; ^3^ Department of Biomedical Magnetic Resonance, Otto‐von‐Guericke University, Magdeburg Germany; ^4^ German Centre for Neurodegenerative Diseases (DZNE), Magdeburg, Sachsen‐Anhalt Germany; ^5^ Centre for Behavioural Brain Sciences (CBBS), Magdeburg, Sachsen‐Anhalt Germany; ^6^ Centre for Clinical Brain Sciences, The University of Edinburgh, Edinburgh, Scotland UK; ^7^ German Center for Neurodegenerative Diseases (DZNE), Magdeburg Germany; ^8^ Department of Neurology, Otto‐von‐Guericke University, Magdeburg Germany; ^9^ Institute of Cognitive Neurology and Dementia Research (IKND), Otto‐von‐Guericke University, Magdeburg Germany

## Abstract

**Background:**

To date, all computerised perivascular spaces (PVS) quantification methods require case‐wise, imaging modality, or study‐specific parameter adjustments, and suffer from generalisability problems in clinical settings, and misdetection of other cerebral small vessel disease (CSVD) markers. We propose a deep learning‐based PVS detection method to overcome these issues. We compare our proposal on magnetic resonance imaging data of CSVD participants against the performance of the Frangi filter.

**Method:**

T1‐weighted (T1w) and T2‐weighted (T2w) neuroimaging data from 16 patients with severe CSVD were collected at the Hospital of the Otto‐von‐Guericke University Magdeburg. PVS across the entire brain tissue were manually segmented in T2w images by a medical student.

We extended SynthSeg (https://surfer.nmr.mgh.harvard.edu/fswiki/SynthSeg) for PVS detection (Figure 1). PVS‐SynthSeg, our proposal, is based on a convolutional neural network and trained using synthetic data sampled from a generative model that relies on brain atlases as a conditioning factor, as well as domain randomisation. We incorporated tubular structures into such brain atlases to expand the model’s capabilities for the identification of PVS. This provides PVS‐SynthSeg with the ability to detect PVS in scans from diverse scanners and protocols without requiring additional fine‐tuning or parameter adjustments.

We compared the precision and recall of PVS‐SynthSeg against that from the Frangi filter on both imaging modalities.

**Result:**

The Frangi filter detected the majority of PVS in both T1w and T2w scans, albeit with high false positive rates–recall of approximately 80% and precision below 30% (Figure 2). PVS‐SynthSeg had more variability in recall but near‐one precision across both modalities, despite the presence of white matter hyperintensities (WMH).

**Conclusion:**

We propose a deep learning‐based PVS detection algorithm. In a small sample, PVS‐SynthSeg detected PVS in T1w and T2w scans better than the Frangi filter without additional adjustments, even in scans affected by motion artefacts or with WMH. These findings highlight the promise of PVS‐SynthSeg as a modular and effective PVS detection method, however larger cohort testing needs to be conducted. Better PVS quantification can help us shed light into the role of PVS in CSVD and Alzheimer’s disease.